# Nanogels as novel drug nanocarriers for CNS drug delivery

**DOI:** 10.3389/fmolb.2023.1232109

**Published:** 2023-08-08

**Authors:** V. Manimaran, R. P. Nivetha, T. Tamilanban, J. Narayanan, Subramaniyan Vetriselvan, Neeraj Kumar Fuloria, Suresh V. Chinni, Mahendran Sekar, Shivkanya Fuloria, Ling Shing Wong, Anupam Biswas, Gobinath Ramachawolran, Siddharthan Selvaraj

**Affiliations:** ^1^ Department of Pharmaceutics, SRM College of Pharmacy, Faculty of Medicine and Health Sciences, SRM Institute of Science and Technology, Kattankulathur, Tamilnadu, India; ^2^ Jeffrey Cheah School of Medicine and Health Sciences, Monash University, Bandar Sunway, Selangor Darul Ehsan, Malaysia; ^3^ Center for Transdisciplinary Research, Department of Pharmacology, Saveetha Dental College, Saveetha Institute of Medical and Technical Sciences, Saveetha University, Chennai, Tamil Nadu, India; ^4^ Faculty of Pharmacy, AIMST University, Bedong, Kedah, Malaysia; ^5^ Department of Biochemistry, Faculty of Medicine, Bioscience and Nursing, MAHSA University, Selangor, Malaysia; ^6^ Department of Periodontics, Saveetha Dental College and Hospitals, Saveetha Institute of Medical and Technical Sciences, Saveetha University, Chennai, India; ^7^ School of Pharmacy, Monash University Malaysia, Bandar Sunway, Selangor Darul Ehsan, Malaysia; ^8^ Faculty of Health and Life Sciences, INTI International University, Nilai, Negeri Sembilan, Malaysia; ^9^ Faculty of Medicine, AIMST University, Kedah, Malaysia; ^10^ Department of Foundation, RCSI & UCD Malaysia Campus, Georgetown, Pulau Pinang, Malaysia; ^11^ Faculty of Dentistry, AIMST University, Kedah, Malaysia

**Keywords:** nanogels, blood-brain barrier, blood cerebrospinal fluid barrier, brain targeting, drug delivery, CNS

## Abstract

Nanogels are highly recognized as adaptable drug delivery systems that significantly contribute to improving various therapies and diagnostic examinations for different human diseases. These three-dimensional, hydrophilic cross-linked polymers have the ability to absorb large amounts of water or biological fluids. Due to the growing demand for enhancing current therapies, nanogels have emerged as the next-generation drug delivery system. They effectively address the limitations of conventional drug therapy, such as poor stability, large particle size, and low drug loading efficiency. Nanogels find extensive use in the controlled delivery of therapeutic agents, reducing adverse drug effects and enabling lower therapeutic doses while maintaining enhanced efficacy and patient compliance. They are considered an innovative drug delivery system that highlights the shortcomings of traditional methods. This article covers several topics, including the involvement of nanogels in the nanomedicine sector, their advantages and limitations, ideal properties like biocompatibility, biodegradability, drug loading capacity, particle size, permeability, non-immunological response, and colloidal stability. Additionally, it provides information on nanogel classification, synthesis, drug release mechanisms, and various biological applications. The article also discusses barriers associated with brain targeting and the progress of nanogels as nanocarriers for delivering therapeutic agents to the central nervous system.

## Graphical Abstract

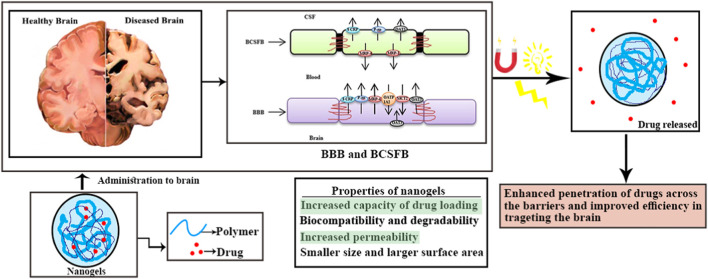

## Highlights

1. Nanogels offer significant advantages over conventional drug delivery systems, overcoming issues like poor stability and low drug loading efficiency.
2. Controlled release by nanogels reduces adverse drug effects and enables lower therapeutic doses, improving patient compliance in various therapies.
3. Nanogels possess key properties such as biocompatibility, biodegradability, and excellent drug loading capacity, making them ideal for biomedical applications.
4. Nanogels show promise as nanocarriers for brain targeting, with the potential to efficiently deliver therapeutic agents to the central nervous system by navigating the blood-brain barrier.

## 1 Introduction

Considering the modern demand for enhancing ongoing therapies and diagnostic examinations, nanotechnology plays a crucial role in developing new and more effective pharmaceuticals (Neamtu et al., 2017). Over the past decade, there has been active research and growth in using nanoparticles as reservoirs for drug and gene transport (Bagheri F et al., 2021). Nanogels are three-dimensional, hydrophilic cross-linked polymers capable of absorbing large volumes of water or physiological fluids (Yadav et al., 2017). Their size is typically less than 200 nm (Bencherif et al., 2009). Nanogels can be injected and have a high surface area in the gel environment, allowing for effective chemical interactions, and they respond quickly to external stimuli (Rashed et al., 2015). Nanogels consist of various polymers, which can be synthetic, natural, or a combination of both, cross-linked by non-covalent bonds, either chemically or physically (Neamtu et al., 2017). Due to their small size, nanogels serve as potential drug nanocarriers, capable of traversing the smallest capillary vessels and penetrating tissues through a transcellular pathway (Ketan et al., 2022).

**FIGURE 1:**
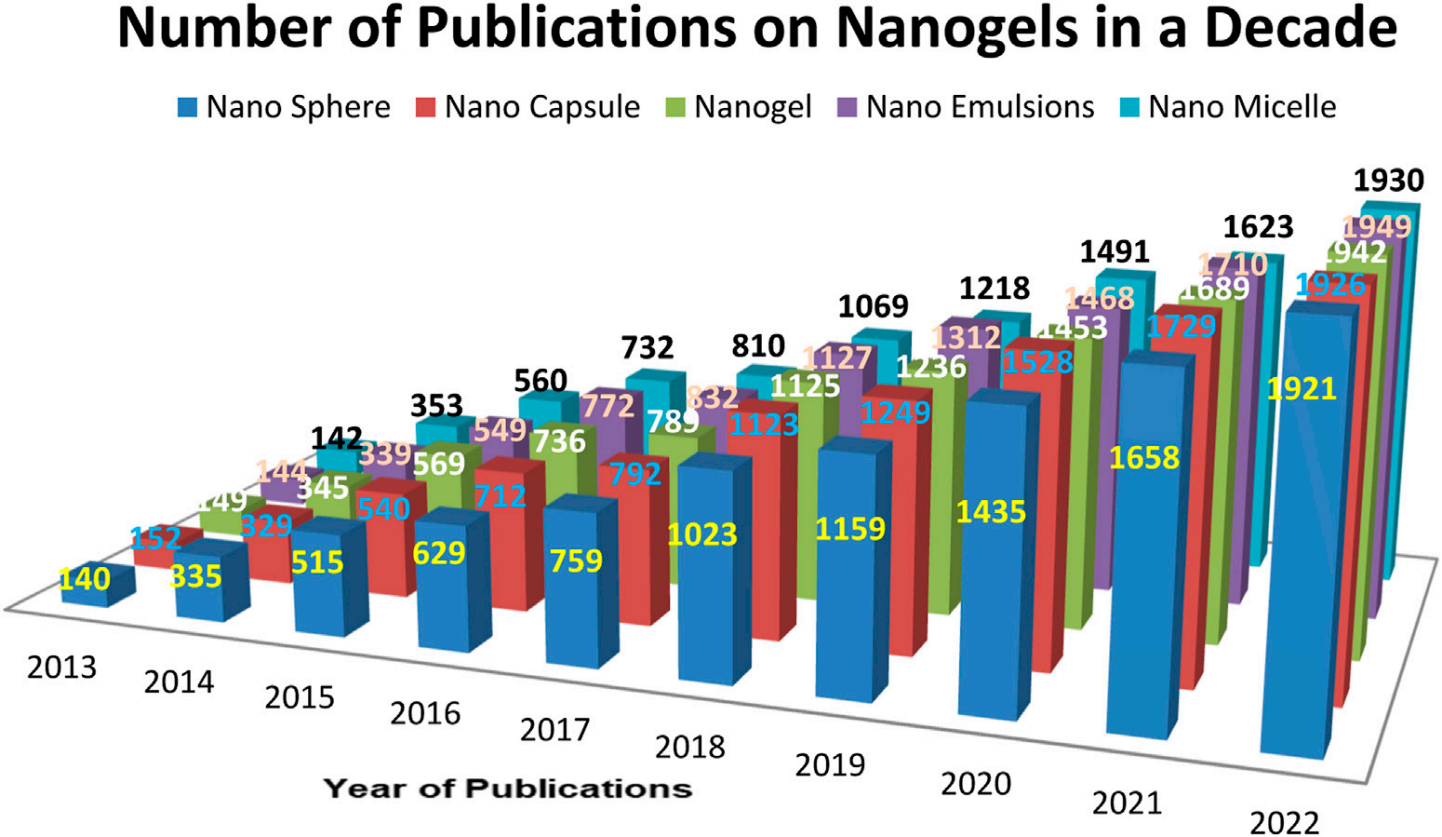
Number of articles published per year based on various Pharmaceutical and polymeric carriers.

In 2020, the Central Brain Tumor Registry of the United States reported an overall primary malignant tumor incidence rate of 7.08 per 100,000 (equivalent to an estimated 123,484 cases), as well as a non-malignant tumor incidence rate of 16.71 per 100,000 cases (Vashist et al., 2018). Currently, a wide range of brain disorders is classified as defects in both psychiatric and neurological aspects, leading to short- and long-term symptoms (Stawicki et al., 2021). The Blood-Brain Barrier (BBB) poses a significant obstacle to drug delivery for any neurodegenerative disorder (Bijuli et al., 2021). Numerous drugs have been discovered for treating various neuronal disorders (Leszek et al., 2017). However, the presence of the BBB and the Blood-Cerebrospinal Fluid Barrier (BCSFB) limits the therapeutic success of these pharmaceutical formulations. These barriers serve as the brain’s biochemical and physiological defense mechanisms (Sharma et al., 2012). The presence of the BBB, along with enzyme interactions and restricted entry of pharmaceutical agents, renders various treatments ineffective (Whiting, 2013). For effective therapy of neuronal disorders, drugs must be highly lipophilic and have a molecular weight ranging from 400 to 600 Da (Wong et al., 2012). Moreover, nanoparticles, in combination with pharmaceutical active ingredients, offer an efficient brain targeting approach for multiple therapies (Alam et al., 2010). Crossing the BBB remains a major challenge in drug delivery. Hydrophilic drugs are transported through specific carrier-mediated endocytosis and the paracellular route, while lipid-soluble drugs are transported via diffusion and P-glycoprotein (Jyoti et al., 2018). Nanogels, known for their non-toxic nature, high drug loading efficiency, and improved BBB permeability, present a promising option (Leonard and Gérard, 2010). The increased investigations of nanogel drug delivery has been noticeable in increased literatures has given the Figure 1. According to 2022, approximately 1942 articles were published until 2022 as a result of increased research into nanogel drug delivery.

Number of articles published per year on various pharmaceutical carriers and polymeric carriers (Database: Scopus, pubmed, science direct. Keywords used for the searches: “Nanogel as drug delivery system” + “polymer” and then each of the following Nanosphere, “nanocapsule”; “nanogel”; “nanoemulsion”, “nanomicelle”).

## 2 Advantages of nanogels

Nanogels offer several advantages over other drug delivery systems for various reasons, including the following:

1. They can target specific sites and regulate the controlled release of bioactive compounds, thereby reducing side effects (Neamtu et al., 2017).
2. By avoiding rapid renal clearance, nanogels exhibit a longer serum half-life (Jain, 2007).
3. Nanogels have an enhanced capacity to encapsulate multiple bioactive compounds in a single formulation (Neamtu et al., 2017).
4. When administered, nanogels remain inactive in the systemic circulation and internal aqueous medium, minimizing the risk of immune response (Stenehjem et al., 2009).
5. Nanogels have a strong affinity for aqueous solutions, enabling them to swell or deswell by absorbing water in an aqueous medium. This characteristic makes them an ideal dosage form for the uptake and delivery of proteins, peptides, bio-macromolecules, and large drugs (Stenehjem et al., 2009).
6. Nanogels are designed to release drugs in a sustained and controlled manner at the desired location, thereby improving therapy outcomes and minimizing adverse effects (Jain, 2007).
7. Nanogels can easily encapsulate both bio-macromolecules and substances of varying molecular weights, allowing for continuous action of these molecules in the biological environment (Leonard and Gérard, 2011).

## 3 Nanogels as novel drug nanocarriers for CNS drug delivery

Due to their special attributes and advantages, nanogels have a large potential for use in the transport of drugs to the central nervous system (CNS). Let’s go into the specifics of how nanogels can be applied to CNS medication delivery:

Versatile and tunable properties: Nanogels are networks of cross-linked polymer chains that are three dimensional and can be created by combining different materials, such as natural polymers (like chitosan or alginate) or synthetic polymers (like polyethylene glycol or poly (N-isopropylacrylamide). Due to their adaptability, their physicochemical characteristics, such as size, shape, surface charge, and mechanical strength, may be precisely controlled. Nanogels can be made to have these characteristics just right for delivering CNS drugs. Nanogels can be made to have these characteristics just right for delivering CNS drugs (Leonard and Gérard, 2010).

1. High drug loading efficiency: Nanogels’ huge surface area and porous structure enable highly effective drug encapsulation. The drug molecules can be physically encapsulated, electrostatically attracted to one another, or chemically conjugated to the nanogel network. The ability to deliver therapeutic doses to the target site is made possible by this high drug loading capacity, which is especially helpful for CNS medication delivery (Jain, 2007).
2. Controlled release of cargo: The ability of nanogels to deliver regulated and prolonged medication release is one of its main benefits. It is possible to design the polymer network of nanogels to react to different stimuli, such as pH, temperature, or enzymes, allowing for the controlled release of the medicine, that is, encapsulated. By reducing systemic toxicity, extending the therapeutic effect in the CNS, and improving medication absorption, this controlled release profile might benefit patients (Jyoti et al., 2018).
3. Enhanced penetration into the blood-brain barrier (BBB): The BBB is a highly selective barrier that prevents most medications from entering the brain, making CNS drug delivery extremely difficult.

Due to their small size and capacity for surface modification, nanogels have demonstrated promise in overcoming this obstacle. The endocytic pathways of brain endothelial cells can be used by nanogels with diameters in the nanometer range to cross the BBB. In order to penetrate the BBB, nanogels with diameters in the nanometer range can take advantage of the endocytic pathways of brain endothelial cells (Stenehjem et al., 2009).

4. Biocompatible and biodegradable: Nanogels’ biocompatibility and biodegradability are essential for the delivery of CNS drugs. Biocompatible materials can be used to create nanogels, assuring low toxicity and compatibility with the CNS environment. Additionally, the use of biodegradable polymers enables nanogels to gradually degrade over time, accelerating their removal from the body and lowering the possibility of long-term buildup (Jain, 2007).
5. Targeted delivery can be achieved: Targeting ligands, such as antibodies, peptides, or aptamers, that precisely recognise and bind to receptors or transporters on the BBB or target cells in the CNS, can be used to surface-functionalize nanogels. These ligands can improve the accumulation of pharmaceuticals at the intended site while reducing off-target effects by facilitating receptor-mediated transport, active targeting, or cellular uptake (Jyoti et al., 2018).

6. Potential for combination therapies: Nanogels’ flexibility enables the encapsulation of many medications inside of a single carrier. Combination therapy are now possible thanks to the ability to deliver the CNS with many medications that each have a unique but complementary mechanism of action. Additionally, by including diagnostic chemicals into their architecture, nanogels can be used to simultaneously diagnose and treat CNS illnesses (Jain, 2007).

The successful application of nanogels as drug nanocarriers for CNS administration, however, necessitates resolving several issues. Ensuring long‐term stability, enhancing drug release patterns, improving brain targeting effectiveness, and addressing potential cytotoxicity and immunogenicity issues related to nanogels are a few of the challenges to be overcome (see Table 1).

**TABLE 1:** Comparison of Nanogels with other Nanocarriers.

Parameters	Nanogels	Liposomes	Polymeric nanoparticles	Solid lipid nanoparticles
Size	Nanoscale	Variable, typically 50–100 nm	Variable, typically 10–200 nm	Variable, typically 10–500 nm
Stability	Good	Moderate	Good	Good
Drug Loading	High	Moderate	High	High
Release Mechanism	Controlled release	Variable	Variable	Variable
Targeting Ability	Can be modified for targeting	Can be modified for targeting	Can be modified for targeting	Can be modified for targeting
Penetration	Can penetrate biological barriers	Can penetrate biological barriers	Can penetrate biological barriers	Can penetrate biological barriers
Biocompatibility	Generally biocompatible	Generally biocompatible	Generally biocompatible	Generally biocompatible
Immunogenicity	Low	Low	Low	Low
Advantages	High drug loading capacity, controlled release	Lipid bilayer mimics cell membranes, can encapsulate a	Versatile drug delivery system with tunable properties	Enhanced stability, improved drug
	ability to target specific sites	wide range of drugs	potential for surface modification	encapsulation
Disadvantages	May require additional steps for synthesis and functionalization	Batch-to-batch variability, limited drug loading ca pacity	Polymeric degradation can lead to loss of stability	May require additional steps for synthesis and functionalization

### 3.1 Limitations of nanogels

The presence of a small amount of surfactant in the nanogel formulation can have a toxic effect if it remains in the body (Jain, 2012). The process of removing solvents and surfactants from the nanogel formulation is both tedious and costly (Jain et al., 2019).

## 4 Properties of nanogels

### 4.1 Biocompatibility and degradability

Nanogels consist of polymers that can be either natural or synthetic. They exhibit high biocompatibility and biodegradability, thereby preventing aggregation in organs. The polymers used in nanogel formulation include chitosan, methyl- and ethyl-cellulose, and various polysaccharides. These polymers are non-toxic, stable, hydrophilic, and biodegradable (Rigogliuso et al., 2012).

Examples: Poly (N-isopropylacrylamide) (PNIPAM), Polyethylene glycol (PEG), Poly (lactic-co-glycolic acid) (PLGA), Polyvinyl alcohol (PVA).

Polymeric micelles: Poly (ethylene glycol)-block-poly (caprolactone) (PEG-b-PCL) and poly (ethylene glycol)-block-poly (lactic acid) (PEG-b-PLA).

### 4.2 Increased capacity of drug loading

Nanogels have a higher drug loading capacity compared to other conventional dosage forms. This is attributed to their superior swelling property, which allows them to absorb a larger amount of water. The loading and incorporation of water provide sufficient space for salts and biomaterials (Soni and Yadav, 2016). Drug loading in nanogels can be achieved through three methods:

• Entrapment by physical means: This involves the linkage between the hydrophilic chains of the polymer and the hydrophobic portion or the dissolution of hydrophobic particles in a hydrophilic carrier (Yadav et al., 2017).
• Covalent attachment of bioactive molecules can result in the formation of a dense, drug-loaded core (Yadav et al., 2017).
• Controlled self-assembly: This method applies to polyelectrolyte-based nanogels, where interaction between oppositely charged electrolytes leads to high loading efficiency (Soni and Yadav, 2016).

Other factors associated with high loading capacity include composition, drug-polymer interaction, and the incorporation of various functional groups into the polymer, as well as molecular weight (Rigogliuso et al., 2012).

### 4.3 Particle size and permeability

Nanoparticles are transported through tissues or endothelium primarily by diffusion. In some cases, they rely on specific carriers, which pose a greater challenge in crossing the Blood-Brain Barrier (BBB) (Rigogliuso et al., 2012). To overcome this obstacle, nanoparticles are purposely manufactured or formulated within a size range of 20–200 nm. Their smaller size enables them to cross the BBB and also helps them evade rapid clearance mechanisms (Jain, 2007).

### 4.4 Non-immunological response

The mononuclear phagocyte system rapidly eliminates foreign substances that enter the systemic circulation through opsonization and phagocytosis. Opsonins bind to the nanoparticle surface, facilitating phagocytic attachment. Opsonization is the process of marking foreign substances to make them visible to phagocytes. Several methods help nanoparticles avoid recognition and remain in systemic circulation for a longer duration. Certain hydrophilic polymers delay the binding of opsonins, making the nanoparticles less noticeable to the immune or defense systems (Rigogliuso et al., 2012).

### 4.5 Colloidal stability

Nanoparticles have a tendency to aggregate, compromising their colloidal stability. To prevent aggregate formation in the bloodstream, nanoparticle formulators modify the surface charge. This can be achieved by increasing the zeta potential (ideally ± 30 mV) to create a stronger repulsion force among particles, resulting in electrostatic stabilization. Incorporating surface modifiers, such as polyethylene glycol (PEG), provides steric effects and hydration forces that contribute to the stabilization of the nanosuspension (Yadav et al., 2017).

Comparing surfactant micelles and polymeric micellar nanogel systems in terms of stability, the latter exhibits greater stability, lower critical micelle concentration, slower dissociation rate, and longer retention of loaded drugs. This is attributed to the higher water content of the polymeric micellar nanogels, which contributes to their dispersion stability (Jain, 2007; Setia et al., 2018).

### 4.6 Nanoscale properties of nanogels

A nanogel is a hydrogel composed of particles in the nanoscale size range. Hydrogels are three-dimensional networks of cross-linked and hydrophilic polymers. Nanogels can swell due to their hydrophilic nature, allowing them to retain large amounts of water.

The utilization of nanogels based on their physical parameters is crucial for a wide range of applications. The size of nanogels, for instance, provides a larger surface area, improving their interaction *in vivo* and facilitating targeted drug delivery for therapeutic purposes (Tobío et al., 1998). Tunability is another important physical property that can be achieved by incorporating amines onto the PEG-PEI surface, making nanogels suitable for treating spinal cord injuries. Employing multi-layer nanogels offers increased stimuli response and enables targeted site-specific action without deformability. Physical characteristics of temperature-sensitive polymers also contribute to enhancing drug loading capacity. Factors such as permeability, thickness, size, storage space, composite covering, and sensitivity to various stimuli influence the stimulus-responsive behavior of nanogels (Kabanov and Vinogradov, 2009; Gonçalves et al., 2010).

Nanogels have emerged as a versatile hydrophilic platform for encapsulating guest molecules that can respond to external stimuli, making them applicable in various fields. These properties not only enhance the functionality of the carrier system but also help overcome many challenges associated with delivering cargo molecules. Nanogels are soft materials with crosslinked networks that can effectively store small molecular medicines, biomacromolecules, and inorganic nanoparticles, enabling their utilization in therapy and imaging for various medical conditions. Despite their evident usefulness, nanogels are not yet widely employed in clinical practice (Vinogradov, 2010).

## 5 Barriers in brain targeting

The brain is protected against the entry of various substances, such as external stimuli, foreign substances, toxins, and pathogens. Homeostasis is maintained through the presence of barriers in the brain, which also regulate the transportation of nutrients, ions, proteins, and metabolites (Vinogradov, 2010). The principal physiological barriers include the following:

### 5.1 Blood-brain barrier

The blood-brain barrier (BBB) was first discovered in 1885 by Paul Ehrlich. It serves as the main barrier separating the brain from the systemic circulation (Apollinariia et al., 2023). The BBB plays a crucial role in protecting the brain from harmful stimuli, infectious agents, and toxins, thereby maintaining homeostasis (Pinelli et al., 2020). Several types of cells are present in the BBB, including astrocytes, brain capillary endothelial cells (BCECs), nerve cells, and pericytes. BCECs are the primary component of the BBB and control the selective permeability of small lipophilic substances. They are connected by tight junctions, which prevent drug transport through the paracellular route. These tight junctions contribute to higher transendothelial electrical resistance (TEER) and prevent the passive diffusion of foreign components from the blood into the brain (Singh et al., 2021; Eva et al., 2023). Pericytes and astrocytes provide support to BCECs and help maintain the function and structure of the BBB (Fakhara Sabir et al., 2019).

The BBB acts as a highly specific semipermeable membrane, allowing only small, low molecular weight, and non-polar components (less than 400 Da) to enter the brain (Kruti et al., 2015). Understanding the structure, function, and nature of the BBB is crucial for the development of various carrier systems designed to deliver drugs to the brain.

### 5.2 Blood-cerebrospinal fluid barrier

The barrier between blood circulation and cerebrospinal fluid (CSF) prevents the entry of drugs, toxins, and other foreign substances. This barrier is composed of arachnoid and choroidal epithelial cells. These cells separate the ventricular CSF and subarachnoidal CSF. The choroid plexus, consisting of choroidal epithelial cells, is the main component of the blood-cerebrospinal fluid barrier (BCSFB) (Gao, 2016). The choroidal epithelial cells are connected through gap junctions, which limit the permeability of the BCSFB. Compared to tight junctions, this junction is less rigid and allows for greater permeability to drugs and certain substances (Patel and M Patel, 2017).

### 5.3 Approaches to nanogel to cross blood brain barrier

1. Surface modification: One method is to add specific ligands or targeting molecules to the surface of the nanogels so that they can interact with the receptors or transporters found on the BBB endothelial cells. These ligands can accelerate the crossing of nanogels by receptor-mediated or active transport processes (Fakhara Sabir et al., 2019).
2. Coating with lipophilic materials: In order to create a lipophilic shell around their hydrophilic core, nanogels can be coated or encapsulated with lipophilic substances like lipid bilayers or surfactants. Through passive diffusion or other transport processes that favour lipophilic molecules, the nanogels may be able to cross the BBB with the aid of this lipophilic coating (Eva et al., 2023).
3. Size reduction: The ability of nanogels to traverse the BBB is greatly influenced by their size. By using mechanisms like receptor-mediated transcytosis or paracellular transport, shrinking nanogels to the nanoscale range can improve their transportation across the BBB (Fakhara Sabir et al., 2019).
4. Use of carrier systems: Nanogels can be included in carrier systems that have been created to cross the BBB, such as nanoparticles or liposomes. These carrier systems can offer nanogels security, stability, and improved transportation through the BBB (Kruti et al., 2015).

It is significant to note that bridging the BBB is a challenging and ongoing research subject, and no single strategy is suitable for all varieties of nanogels. The strategy chosen will depend on the unique characteristics of the nanogels, the intended therapeutic or diagnostic application, and the precise location of the delivery in the brain. Hydrophilic nanogels are being delivered across the BBB more efficiently for effective brain targeting s to the unique methods that researchers are still investigating and developing.

## 6 Nanogel classification

**FIGURE 2:**
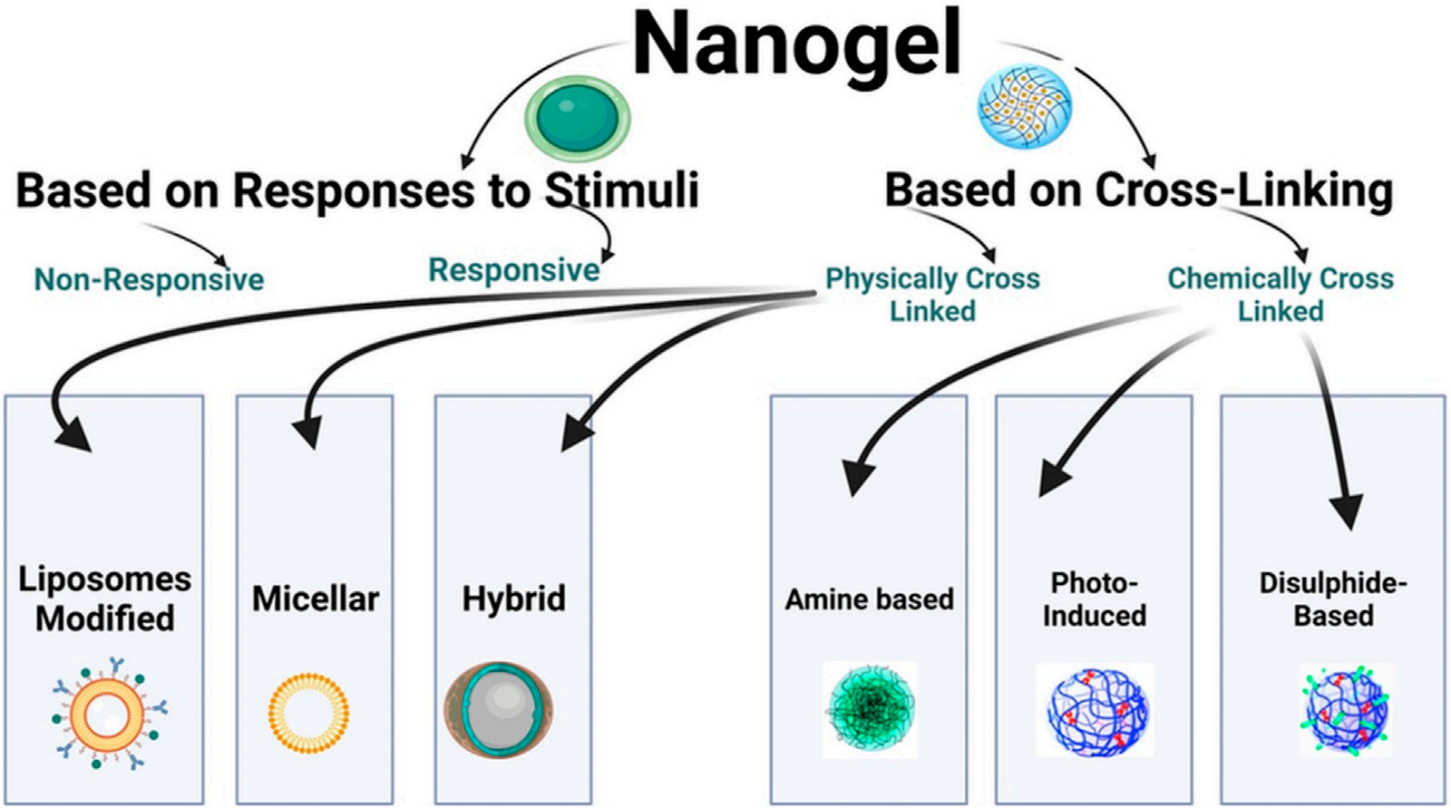
Flowchart illustrating the classification of nanogels.

Figure 2 shows the flowchart illustrating the classification of nanogels.

### 6.1 Based on response to stimuli

#### 6.1.1 Non-responsive

When non-responsive nanogels come into contact with aqueous media, they undergo swelling through simple absorption. This swelling enables the sustained release of drugs at the target site, independent of external stimuli (Reeve et al., 2014).

#### 6.1.2 Responsive

Responsive nanogels can undergo swelling or deswelling in response to various changes in environmental conditions, such as electric current, pH, light, magnetic field, ultrasound, ionic strength, solvent composition, and more (Ajeet et al., 2019). Both synthetic and natural polymers are widely used to formulate stimuli-responsive nanogels, which have a high water absorption capacity for swelling. These nanogels can transition from a swollen state to a collapsed state in response to different stimuli, whether physical, chemical, or biochemical. This transition in nanogels is reversible and occurs rapidly due to the stimuli (Nagpal et al., 2013). Nanogels that exhibit responsiveness to one or more stimuli are referred to as multi-responsive nanogels (Pardridge, 2003).

### 6.2 Based on cross-linking

#### 6.2.1 Physically cross-linked nanogels

They are also known as pseudo-gels, where cross-linking occurs through a physical process and depends on the characteristic features of the polymer, such as composition, temperature, concentration, and the ionic strength of the medium (Yadav et al., 2017). The physical processes involved in cross-linking include van der Waals forces (Soni and Yadav, 2016), hydrogen bonding (Bencherif et al., 2009), electrostatic interactions, aggregation, crystallization, or complexation. The formation of physically cross-linked nanogels can be achieved rapidly using various simple technologies.

##### 6.2.1.1 Liposome-modified nanogels

This type of nanogel acts as an advanced drug carrier that efficiently treats various diseases. These physically cross-linked nanogels are responsive to stimuli and are used as transdermal drug delivery devices due to their unique properties (Yadav et al., 2017). The release of liposomes is influenced by various stimuli, such as pH, temperature, and more. For instance, calcein is effectively delivered to the cytoplasm using liposomes containing succinylated poly (glycerol) at pH 5.5 (Pardridge, 2011).

##### 6.2.1.2 Micellar nanogels

**FIGURE 3:**
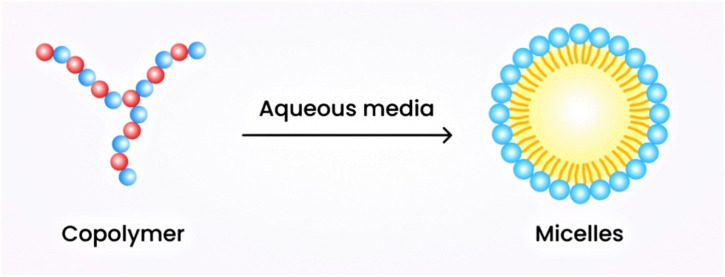
Schematic diagram illustrating micellar nanogels.

These nanogels are formulated through supramolecular self-assembly of hydrophobic and hydrophilic moieties or through the use of graft copolymers in aqueous media. They also feature a core-crosslinking framework (Ribovski et al., 2021). The hydrophilic polymer segment is surrounded by the hydrophobic segment, forming the core. The hydrophilic segment plays a crucial role in hydrogen bond formation, resulting in the stable formation of a core-shell structure that surrounds the micelle. The shell formed is responsive to stimuli and exhibits stability. These nanogels possess a sufficient surface area to accommodate drugs using a simple physical entrapping technique (Shah et al., 2020). The schematic diagram of micellar nanogels is illustrated in Figure 3.

##### 6.2.1.3 Hybrid nanogels

**FIGURE 4:**
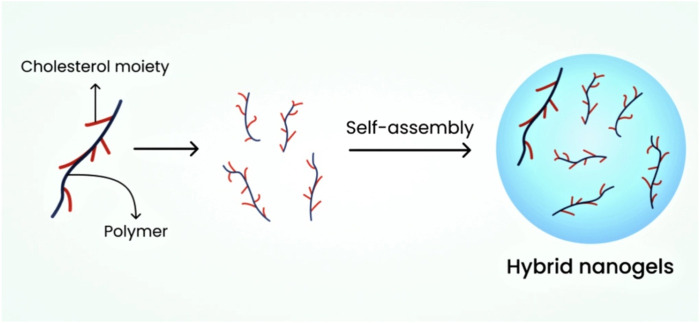
Schematic diagram illustrating hybrid nanogels.

The dispersion of nanoparticles in nano-size in organic and inorganic media results in the formation of hybrid nanogels. Researchers have found that these nanogels can be formulated using aqueous media through polymer self-assembly, which exhibits excellent efficiency in drug release at the targeted site (Dragan and Cocarta, 2016). These nanogels serve as effective drug delivery systems for anti-neoplastic drugs and insulin (Jain, 2007). Figure 4 depicts the schematic diagram of hybrid nanogels.

#### 6.2.2 Chemically cross-linked nanogels

**FIGURE 5:**
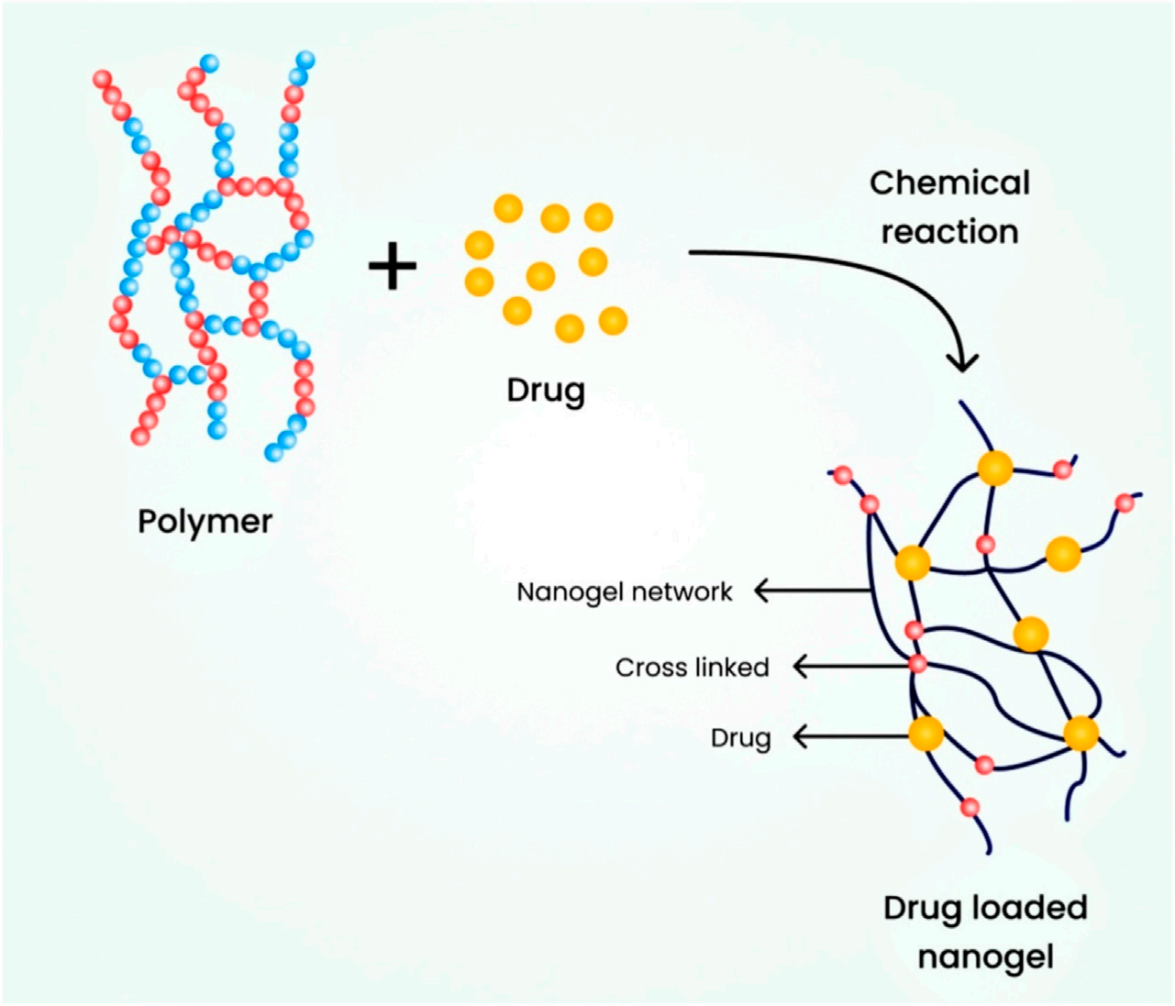
Schematic diagram illustrating chemically cross-linked nanogels.

In this type, nanogels are connected through covalent bonds and chemical linkages. The strength of the linkage greatly depends on the functional groups present in the nanogel molecules. To synthesize these nanogels, also known as cross-linking points, the polymeric nanogels are cross-linked at specific locations. It is essential to prepare nanogels with diverse properties and for broader applications by utilizing different polymers and specific chemical linkages (Yadav et al., 2017). For instance, an environmentally friendly chemical process was used to cross-link polymeric chains with thiol pendant groups, resulting in the development of a nanogel ranging from 20 to 200 nm (Sasaki and Akiyoshi, 2010). Figure 5 illustrates the schematic diagram of a chemically cross-linked nanogel.

##### 6.2.2.1 Photo-induced nanogels

These nanogels can be stimulated and activated through photo-irradiation of photosensitive reactants, which can make them responsive to light. Photo-responsive nanogels can be prepared using this approach. This method has also been used for the synthesis of biodegradable nanogels by inducing salt formation (Almeida and Amaral, 2012; Nosrati et al., 2018). However, this method has limitations due to the cytotoxicity it may produce. Selecting an appropriate photo-initiator is essential to produce highly adaptable and non-toxic gel networks (Nosrati et al., 2018).

##### 6.2.2.2 Nanogels which are amine based

Amine glycoproteins were commonly used for the synthesis of nanogels due to the higher reactivity of activated esters, carboxylic acids, isocyanates, etc., (Thompson and Dua, 2017). The reaction between pentafluorophenyl (PFP) activated esters, which are hydrophobic, and diamine (cross-linker) produces hydrophilic nanogels. The cross-linking degree was optimized by adjusting the amount of cross-linker used and calculating the swelled volume of the nanogels, which should be at least thirty times higher than that of the initial nanogel formulation. Chitosan nanogels were formulated through the reaction of chitosan and dicarboxylic acid amine groups in a water-in-oil microemulsion (Hasegawa et al., 2009).

##### 6.2.2.3 Disulphide-based nanogels

The fabrication of redox-sensitive nanogels is achieved by incorporating disulfide bonds in the cross-linking structure, coordinated with a metal ligand along with a hydrophilic polymer. Metal-based ligands are utilized in the formulation of these nanogels. Redox-responsive nanogels offer advantages in gene delivery applications (Sahu et al., 2017).

##### 6.2.2.4 The role of shape and size of Nanogels

A three-dimensional crosslinked polymer network makes up nanogels, which are nanoparticles. They are adaptable nanomaterials with tunable properties that are suitable for a wide range of applications, such as surface coating and targeted drug delivery. The functionality and behaviour of nanogels are greatly influenced by their shape, size, and surface charge.

##### 6.2.2.5 Shape

Nanogel behaviour, stability, and interactions with biological systems are strongly influenced by their shape. Depending on the fabrication techniques used, nanogels can be designed in a variety of shapes, such as spherical, rod-like, or disk-like. The surface, surface charge distribution, and interaction with target cells or tissues can all be affected by the shape.

A particular sort of nanogel that has a high surface area to volume ratio and can effectively encapsulate therapeutic compounds is spherical nanogels, which also have a higher drug loading capacity. Due to their elongated shape, rod-like nanogels can exhibit improved cellular uptake, whereas disk-like nanogels can offer improved stability and control over release kinetics (Patel et al., 2019).

##### 6.2.2.6 Size

Nanogels are typically used for these applications and typically come in sizes between a few nanometers and a few hundred nanometers.

Depending on the precise use and desired properties, the size range of nanogels used for surface coating and targeted delivery can change. Smaller nanogels (diameters between a few and 50 nm) typically have advantages like improved stability, effective therapeutic molecule encapsulation, and simple cellular uptake. Nanogels that are between 50 and 200 nm in size are a good compromise between stability, drug-loading ability, and circulation time. Extended circulation and enhanced stability can be provided by large nanogels (those larger than 200 nm).

The behaviour and functionality of nanogels can be influenced by their size. The desired drug-loading capacity, the release kinetics, the interaction with the target tissues or cells, and the route of administration all have an impact on the selection of size range. Researchers can optimise the performance of nanogels for particular applications, such as surface coating and targeted delivery, by carefully controlling their size (He et al., 2011).

##### 6.2.2.7 Surface charge

Nanogel stability, cellular interactions, and targeted delivery are significantly influenced by the surface charge of nanogels, which is determined by the presence of charged functional groups on their surface. When making nanogels, charged monomers can be added, or charged molecules can be applied to change the surface charge.

The electrostatic repulsion or attraction of nanogels is influenced by their surface charge, which can either prevent or cause aggregation. Additionally, the interaction of nanogels with biological membranes and cellular uptake can be modified by the surface charge of the materials. For instance, because they can interact with negatively charged cell membranes, cationic nanogels frequently show improved cellular uptake. Anionic nanogels, on the other hand, might have less nonspecific binding and a longer bloodstream circulation time (Kulkarni et al., 2019).

Researchers can modify the surface charge, size, and shape of nanogels to tailor their properties for particular uses. These parameters, for instance, can be adjusted to achieve effective encapsulation, controlled release, prolonged circulation, and targeted delivery to particular tissues or cells when it comes to drug delivery. Utilising nanogels’ potential for biomedical applications critically depends on designing them with the desired shape, size, and surface charge.

## 7 Nanogel synthesis

### 7.1 Photolithographic technique

It is a novel technique that provides strict control over nanogel particle size, morphology, and composition, and enables the loading of bio-macromolecules and pharmaceutical drugs. This technique utilizes photochemical reactions for activation with the aim of producing three-dimensional nanogels for drug delivery. Molds and stamps or replicas are treated with this method, allowing for the release of incorporated agents from the molded gels (Wang et al., 2010). The release or adhesion of molded gels to the substrate can be enhanced through surface modification.

**FIGURE 6:**
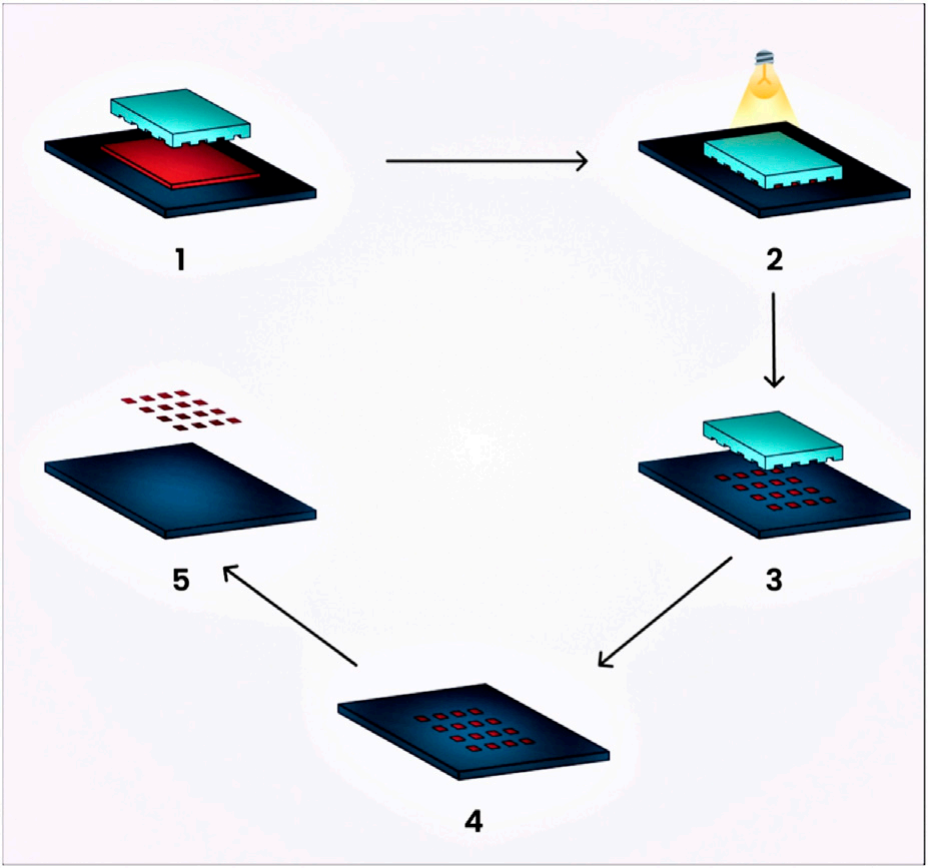
Schematic diagram illustrating the preparation of nanogels using the photolithographic technique.

This technique consists of five stages:

1. The low surface energy polymer, which is UV cross-linkable, acts as a substrate and is released onto the prebaked, photoresist-coated water (Chacko et al., 2012).
2. The next step involves molding the polymer into shapes on the silicon wafer by pressing the polymer with the quartz base and exposing it to extreme UV light (Chacko et al., 2012).
3. Extracting the quartz exposes the particles, which consist of a thin residual interconnecting film layer (Chacko et al., 2012).
4. This thin layer is eventually dissolved by plasma that contains oxygen, oxidizing it (Chacko et al., 2012).
5. The fabricated particles are obtained immediately through the dissolution of the substrate in a water buffer (Chacko et al., 2012). A schematic diagram of the preparation of nanogels by the photolithographic technique is represented in Figure 6.

The prominent benefits of this technique include its ability to produce non-spherical nanogels. Such non-spherical particles can experience a longer circulation period in the human body because spherical particles are more prone to phagocytosis compared to ellipsoidal or disc-shaped particles (Zhuang et al., 2012). Standardized particle size is crucial for drug delivery using nanogels, as the distribution of nanogels throughout the body and their interaction with cells can be influenced by particle size (Pujana et al., 2012).

### 7.2 Modified pullulan technique

**FIGURE 7:**
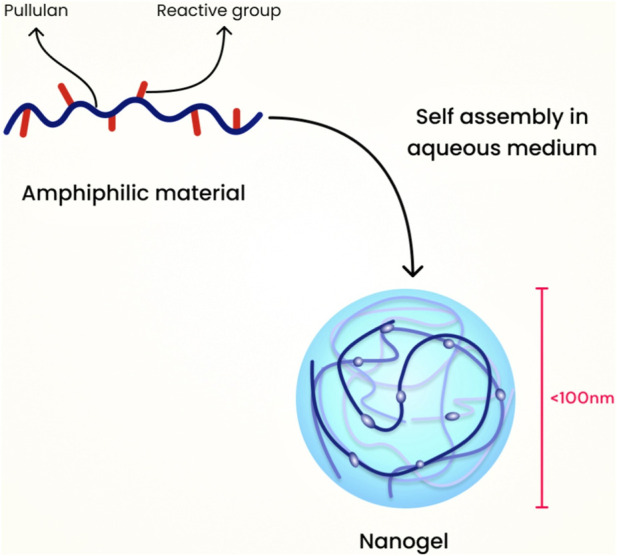
Schematic diagram of nanogel preparation using the modified pullulan technique.

For this category, an example is the hydrophobic self-assembled pullulan nanogel. The pullulans are modified in phases, starting with the use of methacrylate, followed by the incorporation of hydrophobic 1-hexadecanethiol. The resulting material is amphiphilic, and it assembles through hydrophobic interactions between the alkyl chains when water is added. Pullulan serves as a robust protein carrier and has been used in nanogel formulations for drug delivery (Sasaki et al., 2011). Figure 7 illustrates the schematic diagram of nanogel preparation using the modified pullulan technique.

### 7.3 Emulsion polymerization technique

**FIGURE 8:**
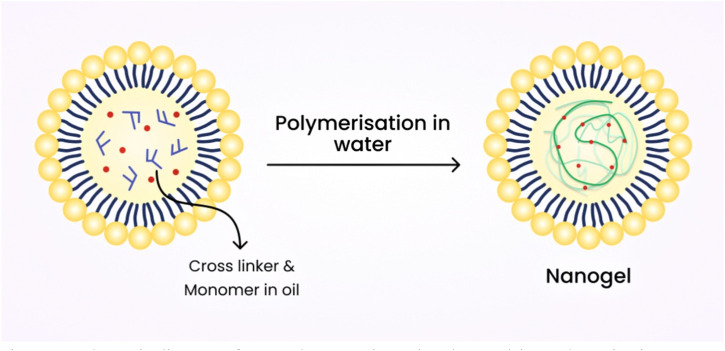
Schematic diagram of nanogel preparation using the emulsion polymerization technique.

Usually, nanogels that are chemically cross-linked are produced from water-soluble cross-linked polymers, such as modified polysaccharides, using reactive compounds like vinyl and thiol groups under diluted conditions. Emulsion polymerization methods have been commonly employed to produce nanogels with excellent control over their size. Nanogels are formed through polymerization in the presence of surfactants within O/W nano or microemulsions (Matsumoto et al., 2009). Figure 8 shows the schematic diagram of nanogel preparation using the emulsion polymerization technique.

Nanogels of L-proline functionalized poly (methyl methacrylate) (PMMA), as well as catalyst functionalization range (0.5–15 wt% and 0–50 wt%) as densities of cross-linking were formulated by this technique (Zhuang et al., 2012). Monomer droplets are formulated by this technique via mechanical stirring (TaleiFranzesi et al., 2006).

### 7.4 Inverse miniemulsion polymerization technique

**FIGURE 9:**
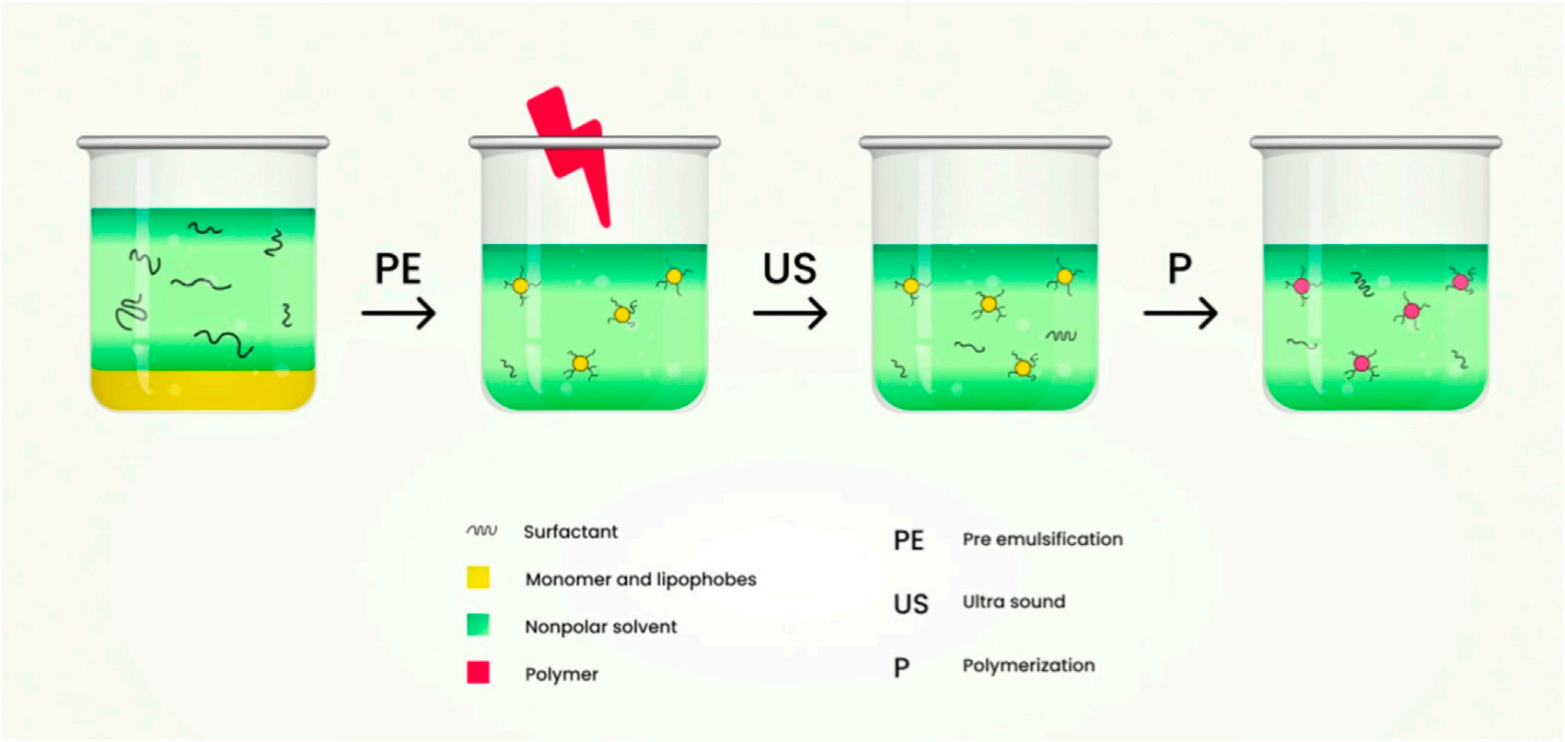
Schematic diagram of the preparation of nanogels by inverse miniemulsion polymerization technique.

This technique consists of the dispersed phase which is polar in nature and a continuous phase with lower polarity. The dispersed phase which is polar in nature is usually stabilized sterically using surfactant. The hydrophilic-lipophilic balance (HLB) value of the surfactant must be low to provide colloidal stabilization. Stable inverse miniemulsions are produced by either a homogenizer or a mechanical high-speed stirrer under high shear. For the dispersed phase to limit the molecular net diffusion, a co-stabilizer (generally hydrophilic salts) is needed. Co-solvents like water, dimethyl sulfoxide (DMSO) may often be added to the dispersed phase to enhance the monomer’s solubility and to facilitate salt dissociation to boost the stability of droplets (K Oh et al., 2008). The schematic diagram of the preparation of nanogels by inverse miniemulsion polymerization technique is shown in Figure 9.

The activator-induced electron and atom transfer radical polymerization of oligo (ethylene oxide) monomethyl ether methacrylate via inverse miniemulsion polymerization of cyclohexane/water at room temperature formulates fluorescent-labelled or fluorescent dye Rhodamine B nanogels. The HO-POE0300MA functional nanogels were prepared with atom transfer radical polymerization (ATRP) containing hydroxyl initiator to control polymerization. During the polymerization, nanogels which are adhesive to the cells is synthesized using ACRLPEO-GRGDS as a co-monomer (Bencherif et al., 2009). Monomer droplets are formed in oil in water (O/W) miniemulsion process by applying high shear stress using ultrasonication or a homogenizer under high pressure. It is kinetically stable (TaleiFranzesi et al., 2006).

### 7.5 Reverse microemulsion polymerization technique

This technique is used to formulate nanogels filled with lithium polyacrylic acid (PAA). Span 80 weighing 3.43 and 2.62 g was added to 100 ml of hexane that acts as an oil phase and was stirred by a magnetic stirrer; 10% w/w lithium hydroxide (LiOH) (10 ml) in water was added to 500 μl acrylic acid and this serves as an aqueous phase. To the aqueous phase add 5% w/v of N, N′-methylenebisacrylamide (MBA) suspension (214 μl), 20% w/v of N, N, N′, N′-tetramethylenediamine (TEMED) (40 μl) and 2% w/v of potassium persulphate (500 μl).

**FIGURE 10:**
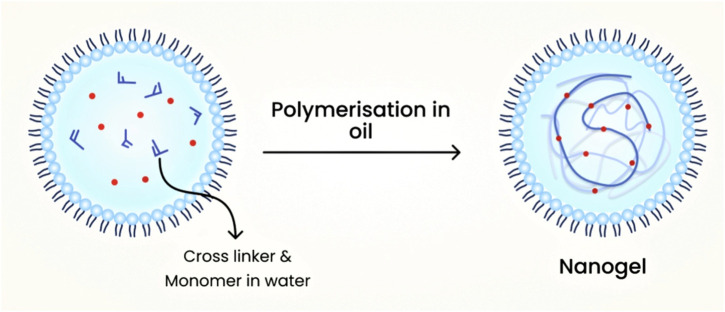
Schematic diagram of nanogel preparation by reverse microemulsion polymerization method.

Microemulsions were prepared by the addition of aqueous medium into oil medium drop wise. The formed emulsion was transferred to water bath of 60°C by using magnetic stirrer. It was stirred at 400rpm and held overnight at room temperature. The pellets were collected after decanting the supernatant and the formed microemulsion was thermodynamically stable (Sultana et al., 2013). This technique contains aqueous droplets, sustainably dispersed in a continuous organic media with the assistance of a significant amount of surfactants which is oil-soluble; polymerization takes place within the aqueous droplets, creating robust hydrophilic nanogels which are colloidal in nature with a diameter of 50–100 nm. Schematic diagram of nanogel preparation by reverse microemulsion polymerization method is shown in Figure 10.

### 7.6 Free radical cross-linking polymerization technique

In one reaction, conventionally uncontrolled free radical cross-linking monomer copolymerization blends the two mechanisms of copolymerization as well as cross-linking. The reactions take place in the droplets which are nanosized produced by surfactant as well as the co-surfactant which is sufficient for the eventual reaction of developing monomer micelles as mini-reactors. The size and stability of the nanogels formed eventually depend on the parameters involved in the reaction like the quantity and type of surfactant utilized and the shear stress duration. The key feature is to prevent macro-gelation by “templating” approaches focused upon heterogeneous polymerization in colloidal conditions like polymerization of mini- and microemulsion, or dispersion and precipitation. These approaches with merits and demerits are discussed in Table 2.

**TABLE 2:** Free radical polymerization technique for the synthesis of nanogels with merits and demerits.

Reaction	Description	Merits	Demerits	Reference
Miniemulsion	Production of droplets which are nanosized by higher shear stress of monomer and surfactant mixtures	Size distribution is narrow within the range of 50–100 nm	• The fundamental use of special equipments like ultra sonicator	Petros and DeSimone (2010)
			• Requirement of surfactant and co-stabilizer	
Microemulsion	• High shear stress is absent	• Production of nanogels in the size range of 10–150 nm	• Surfactant concentration is higher	Menkie et al. (2017)
	• Monomers are in the form of micelles	• Absence of shear stress	• Co-surfactant use is required	
Dispersion	• All the reaction components are soluble within the reaction medium	The size ranges from 0.1 to 15 mm and it is adjusted with that of the concentration of monomer and the dispersing agent	Superior choice for vinyl functionalized monomers	Menkie et al. (2017)
	• The process of polymerization occurs in the homogeneous stage			
	• The polymers used are insoluble and with the help of colloidal stabilizing agents produce a robust dispersion			
Precipitation	• Induction of the reaction appears in the homogeneous stage of the monomers	• There is no requirement of surfactant	Often irregular in shape	Menkie et al. (2017)
	• The particles are separated by the mechanism of cross-linking	• The size ranges from 100 to 600 nm and it is adjusted using concentration of the monomer		

The nanogels which are photocrosslinked biodegradable photoluminescent polymers (PBPLPs) were designed for the delivery of drug and cell imaging through this method and this involves the cross-linking of vinyl-containing fluorescent prepolymer. Production of these nanogels shows a new age for the production of nanobiomaterials for the delivery of drug and cell imaging in theranostic nanomedicine (Ferreira et al., 2011).

## 8 Drug release mechanism of nanogels

In order to boost the concentration of the drug at the site of action the drug delivery mechanisms are typically intended to transport the drug to the site and to furnish controlled release. The nanogels tend to discharge the drug rapidly in the form of a burst release and this contributes to the depletion of hydrophilic and hydrophobic medications in circulation directly after administration. There is already a small volume of drug which is intact inside the nanogel and can reach the target position (Leonard and Gérard, 2011; Lu et al., 2013). The release of the medication to the intended site can be controlled with the aid of polymer which slows down the release from nanogel (Lu et al., 2013). The drug release kinetics is regulated entirely by swellability, polymer type, viscosity, consistency, etc. These systems have been developed primarily to achieve controlled drug release. So, their aim is to achieve kinetics of zero order. Since nanogels are thermodynamically as well as kinetically robust, these structures are supposed to display prolonged release properties.

The medications from the nanogels can be released through various mechanisms such as:

1. Diffusion

The polymeric micelles in nanogels employing the mechanism of diffusion have shown the abilities to reduce the toxicity profile, enhancement of targeted drug delivery, improvement in the therapeutic profile of the active constituents. Application of diffusion process for delivery of therapeutic agents possess disadvantages which includes blood components interfering with the micelle formation showcasing difficulty in correlating stability profile of in vivo-in vitro characteristics. Also, the measurement of the sustained release compounds obtained from micelles are demanding for evaluation (Sanson and Rieger, 2010; Cao and Ziener, 2013).

2. Degradation

The release of drug due to nanogel degradation can be employed to formulate drugs such as Rhodamine 6 and Doxorubicin. The advantages of utilising this mechanism are improved drug release characteristics because of the usage of pH responsive character of the nanoparticles. The degradation mechanism helps in the release of nanocomplexes which will be helpful in overcoming the drug resistance seen in cancerous cells. The limitations of utilisation of degradation mechanism is the employment of single polymer for degradation which constrains the drug responsiveness in the environmental complexity of the tumour tissues (Crespy and Landfester, 2010; Larsson et al., 2014).

3. pH shift

For the mechanism of drug released based on pH responsiveness, the release is determined by the differential change in the pH value during the time course of a disease. The advantage lies in the fact that there nanogels shows targeted drug delivery to pH specific regions and also regulates the release rate of drug depending on the environmental changes. The disadvantages of utilising pH responsive nanogels is the high cost of polymers employed e.g., Polyelectrolytes. Alongside the cost of formulation, the manufacture of such agents requires tedious process and the circulation, targeting through blood vessels is arduous due to its higher size (Manry et al., 2011; Sagar et al., 2018).

4. External energy like magnetic field, light etc.

An example of mechanism of drug release from nanogel based on environmental cues or signals is the development of stimuli responsive nanogel drug delivery system known as SRDDS. They have the advantages which incorporates increased responsiveness to the environmental stimuli or microenvironmental differences and are capable of releasing drugs at the targeted site. The environmental cues can be based on temperature, pH etc., which plays a major role in the drug release rate. The demerits of using photo sensitisers as release mechanisms is its hydrophobic characteristics along with lower bioavailability (Ahmed et al., 2020).

Thermosensitive nanogels used for targeted drug delivery employs temperature as a criterion for drug release. These gels possess the benefit of bypassing first pass metabolism since this type of formulation can be directly injected into the bloodstream. They provide with an uniform flow of the drug along with a controlled drug release by avoiding burst release of the therapeutic agent. The limitations of the thermosensitive nanogels are the poor biocompatibility, a gradual response to variations in temperature and subdued mechanical properties of the polymers employed (Croy and Kwon, 2006).

**FIGURE 11:**
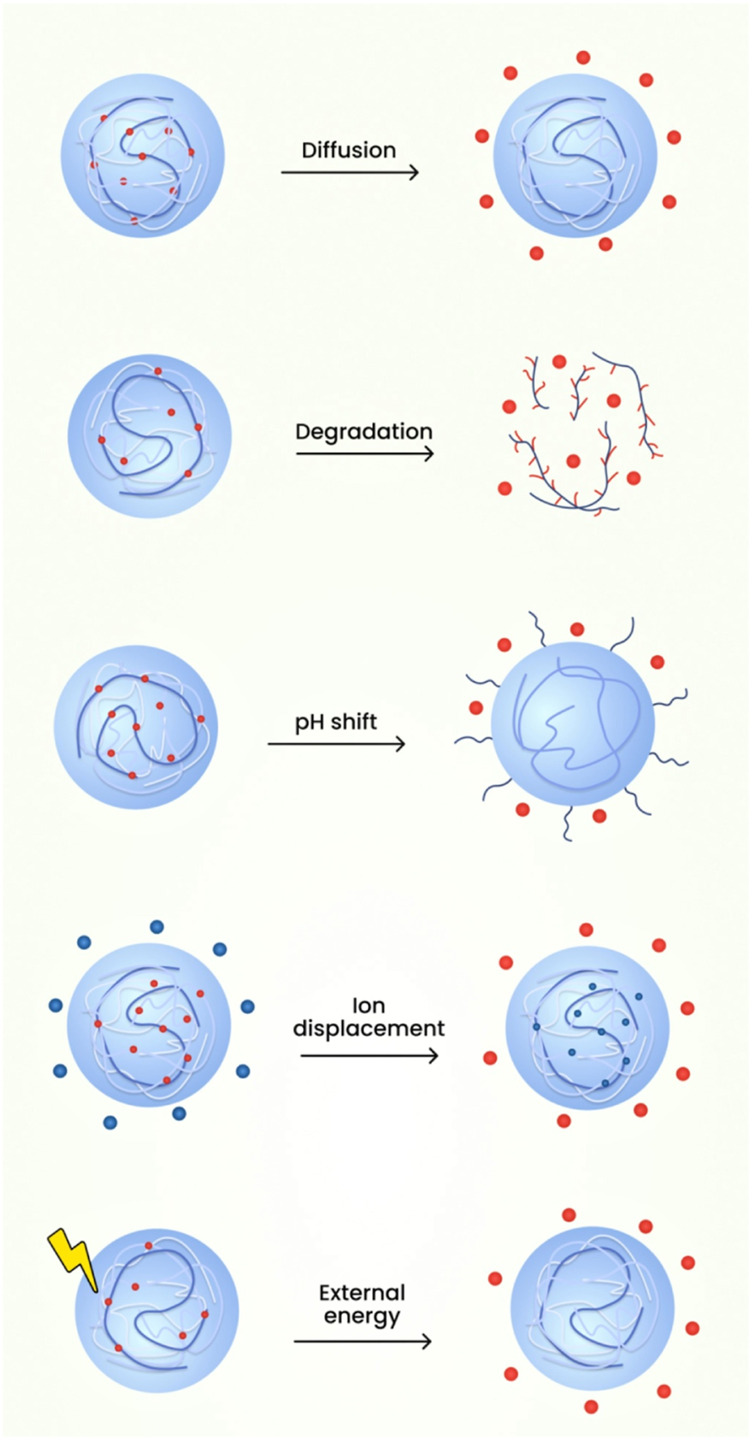
The schematic diagram of the various mechanisms involved in the drug release from nanogels.

The recent evidences shown that the near-infrared (NIR) light in wavelengths range of 650–900 nm has been considered to stimulus to trigger drug release. The main advantage of this drug release includes mild reaction conditions, higher bio compatibility, lower toxicity, less usage of organic solvents and lower by-product formation. This photoreaction can also be reversible. NIR radiation can also penetrate more than 10 cm into tissue under specific circumstances. Photo-responsive polymeric nanogels often contain acrylic or coumarin-based bonds that cleave under illumination causing drug release (Kabanov and Alakhov, 2002; Gonçalves et al., 2014). Reactions or the cleavage of chemical bonds in the nanogel structure shown to produce controlled or sustained drug release. The nanosized property of the carriers provides them with a specific surface area and inner space, which increases the stability of loaded drugs and prolonging their circulation time in biological systems (Subramaniyan et al., 2022). Through the design of specific chemical structures and different methods of production, nanogels can realize diverse responsiveness (temperature-sensitive, pH-sensitive and redox-sensitive) and enable the stimuli-responsive release of drugs in the microenvironments of various diseases (Soni and Yadav, 2016). The schematic diagram of the various mechanisms involved in the drug release from nanogels is shown in Figure 11.

## 9 Other biological applications of nanogels

### 9.1 Local anaesthetic action

These are the classes of drugs which produce analgesic action and eliminate pain. Overdosing of these drugs contributes to higher toxicity and this leads to the formulation of controlled delivery of drug which overcomes the disadvantage. For the improvement of localized administration of the drug they can be formulated as nanogels. Example: Nanogel of methacrylic acid-ethyl acrylate copolymer is loaded with procaine hydrochloride (Anupama and Priyanka, 2018).

### 9.2 Treatment of autoimmune disorder

The inclusion of immunosuppressive drugs used for the delivery of nanogels has been widely explored because nanogels can enhance the impact of immunosuppression via accumulation of drug to the target cells (Yadav et al., 2017).

### 9.3 Treatment of inflammatory disorders

Nanogels are widely used as nonsteroidal anti-inflammatory drugs (NSAIDs) for psoriasis treatment and other contact dermatitis because of their enhanced contact time to the target site. Various anti-inflammatory drugs are formulated in the form of nanogels due to their effectiveness in the deep permeation of skin layers (De et al., 2009).

### 9.4 Treatment of cancer

Treatment of cancer involves delivering the medication to the intended site with the hope that it produces minimal adverse impact to the non-cancerous cells and produces greater therapeutic effect to the tissue which is infected (Tomatsu et al., 2011). The formulation of doxorubicin loaded into acetylated chondroitin sulphate nanogel is used in the treatment of cancer (Samuel et al., 2021).

### 9.5 Transdermal delivery of drugs

This route of administration possesses various advantages because it bypasses hepatic first-pass metabolism, steady-state concentration of the drug in the plasma, enhanced patient compliance, etc. The nanogels formulated for transdermal delivery produces effective delivery of the drug to the stratum corneum. The formulation of aceclofenac in the form of nanogel overcomes the oral administration side effects like gastric bleeding and ulcer and shows greater stability and enhanced permeability (Huang et al., 2019).

### 9.6 Nanogels for microbial infections

The resistance produced by the microorganism for conventional antibiotics is a challenging task for the treatment of infections. The nanogels provide a localized and quick action which is required for the treatment of microbial infections. Example: Dextran which is cross-linked with polyacrylamide nanogel when loaded using zinc nitrate acts as an antibacterial agent (Tan et al., 2010).

### 9.7 Treatment of diabetes

The nanogels which are self-operating are used in the insulin delivery for the treatment of diabetes. Single-dose nanogels which are injectable are formulated and this provides balance of sugar level for at least 10 days. They are sensitive to the alteration in the level of glucose in the systemic circulation and deliver a specific quantity of insulin (Shah et al., 2012).

## 10 Newer trends in drug delivery to the CNS by nanogels

As a better and newer solution to the treatment and diagnosis of a large array of diseases, nanogels have been shown to offer greater advancement in this field. They are used for brain tumour treatment and also in tissue engineering, thus they serve as an ideal nanocarrier for delivering drugs to the CNS (Kaur et al., 2014).

### 10.1 Immunotherapy for brain tumours

Hydrogels incorporated with T-lymphocytes were prepared with poly (ethylene glycol)-g-chitosan (PC gel) for the targeted delivery to the glioblastoma cells for immunotherapy of brain (Mrityunjoy et al., 2017). These nanogels showed greater effectiveness in comparison with matrigel regulation in destroying glioblastoma cells, suggesting the feasibility of such strategy for glioblastoma localized immunotherapy. This research demonstrated PC gel’s cellular compatibility with the T-lymphocytes, which when encapsulated inside the PC gel maintains their antiglioblastoma function. Immunotherapy activates the immune response, which directly requires tumour destruction and rejection with negligible damage to underlying tissues; and it is a promising path to next-generation brain cancer care nanomedicine (Phatak and Praveen, 2012).

### 10.2 Methotrexate-loaded nanogel for brain delivery

Chitosan-based nanogels are well known for their biosafety profile and non-immunogenicity profile. Hydrophilic anticancer agent methotrexate demonstrates low permeability in the BBB. The polysorbate nanogel80 surface functionalization has been studied by inducing the receptor-mediated endocytic cascade of lipoprotein with low-density endothelial cells present in the brain (Malzahn et al., 2014).

### 10.3 Nanogels for the treatment of Alzheimer’s disease

Using self-association in aqueous solution method, cholesterol-bearing pullulans containing polysaccharide backbone and cholesterol molecules which are hydrophobic in nature were formed as a nanogel with a diameter of 20–30 nm. These nanogels have been used to inhibit amyloid β (Aβ) fibril formation which is significant in Alzheimer’s disease pathology (Aimetti et al., 2013). The hydrogels which are amphiphilic diblock co-polypeptide have been investigated as depots which are injectable, which provides sustained delivery of the proteins which are bioactive; and which have an impact on local neurons present in the CNS inside BBB (TsaoKievit et al., 2014).

Due to the existence of blood spinal cord barrier (BSCB), which averts the movement of medications systemically delivered through spinal cord, injectable thermo-sensitive hydrogels or nanogels have gained interest in spinal delivery through intrathecal space (P Restifo et al., 2012).

### 10.4 Delivery of proteins and peptides in the form of nanogels

Incorporation of proteins to nanogels exhibits enhanced therapeutic activity than that of the unmodified nanogels; such nanogels brought advantages for the delivery of proteins and peptide therapeutics which is too large for crossing the BSCB in nature (Azadi et al., 2012).

### 10.5 pH/temperature-sensitive nanogels for diagnosis of glioma

Natural polymer-based nanogels fall into substrate group which is appropriate for various CNS applications. Their hallmark property includes their biodegradability, rigidity, surface compatibility and porosity with CNS tissue and mouldable characteristic feature with functionalization and other alterations. For preoperative diagnosis of glioma, the nanogels’ vulnerability to pH and/or temperature was examined using MRI (Ikeda et al., 2006).

### 10.6 Inhibition of efflux transporters for brain delivery

The biggest challenge with drug distribution is effective transportation of the drug to the target site. The cargo must overcome several barriers before arriving at its target site (Song et al., 2012). Thus, for the treatment of neurological disorders in the CNS, advanced nanocarriers need to be investigated to effectively deliver drugs to the brain (J Begley, 2004). Several studies have demonstrated different techniques for inhibiting the transporters with efflux mechanism demonstrated in BBB with the help of pluronic block copolymers, which possess the ability for the substrate delivery to the brain (M Matsumoto et al., 2013).

### 10.7 Transportation of oligonucleotides

Nanogel-based systems have demonstrated efficient encapsulation and binding of the oligonucleotides which are negatively charged, creating a stable dispersion of polyelectrolyte complex with less than 100 nm particle size which is aqueous in nature (Jiang et al., 2013). The efficient transport across the BBB of oligonucleotides capsulated in nanogels has been demonstrated by an *invitro* model using polarized monolayers of bovine brain microvascular endothelial cells (BMVECs) (Batrakova et al., 2001). The interaction between the polymers and the BMVEC membrane proteins produces improved delivery of the drug therapeutics to the brain (Ketan et al., 2022).

## 11 Conclusion

This review focuses on the nanogels used as a nanocarrier for targeting the brain. As a new and enhanced strategy for the diagnosis and treatment of a wide spectrum of diseases, nanogels possess enormous progress in this field. The three-dimensional hydrogels and nanosized particulate networks merge to gain maximum therapeutic potential in comparison to conventional carriers. This gave rise to the various applications of nanogels. They hold prominent features and circumvent various formulation challenges like drug loading, stability, efficiency and compatibility that render them to be superior to other carrier systems. They also can mitigate the possible side effects of the medication and their therapeutic dose, leading to enhanced therapeutic agent effectiveness and increased benefits for the patients. Specific studies have shown remarkable results that have laid a foundation for the drug molecule delivery which is used for the treatment of various ailments like autoimmune disorders, inflammatory disorders, cancer, diabetes, etc. The new generation of nanogel formulations could monitor the prolonged and selective delivery of drugs in a specific manner. This review provides a systematic framework that highlights the design; barriers associated with brain targeting; and newer trends that need to be addressed in order to realize the maximum capability of drug delivery to the brain as nanogels. Various infectious disorders associated with brain can be effectively treated with the nanogel drug delivery system. Thus, they are considered to be an adaptable carrier system with considerable capability and could offer any kind of advancement for future therapy of various diseases.

## Abbreviations

CNS, central nervous system; BBB, blood brain barrier; BCSFB, blood cerebrospinal fluid barrier; PEG, polyethylene glycol; BCECs, brain capillary endothelial cells; TEER, transendothelial electrical resistance; CSF, cerebrospinal fluid; PFP, pentafluorophenyl; UV, ultra violet; PMMA, poly (methyl methacrylate); HLB, hydrophilic-lipophilic balance; DMSO, dimethyl sulfoxide; ATRP, atom transfer radical polymerization; O/W, oil in water; LiOH, lithium hydroxide; MBA, N, N′-methylenebisacrylamide; TEMED, N, N, N′, N′-tetramethylenediamine; PBPLPs, photocrosslinked biodegradable photoluminescent polymers; NSAIDs, nonsteroidal antiinflammatory drugs; BSCB, bloodspinal cord barrier; BMVECs, brain microvascular endothelial cells.
